# Development of Iron Oxide Nanochains as a Sensitive Magnetic Particle Imaging Tracer for Cancer Detection

**DOI:** 10.1021/acsami.5c00332

**Published:** 2025-03-26

**Authors:** Panangattukara Prabhakaran Praveen Kumar, Md Nafiujjaman, Ashley V. Makela, Kay Hadrick, Meghan L. Hill, Maggie Lee, Taeho Kim

**Affiliations:** Department of Biomedical Engineering, Institute for Quantitative Health Science and Engineering, Michigan State University, East Lansing, Michigan 48824, United States

**Keywords:** magnetic particle imaging, contrast agents, SPION, iron oxide nanochain, magnetic moment, breast cancer imaging

## Abstract

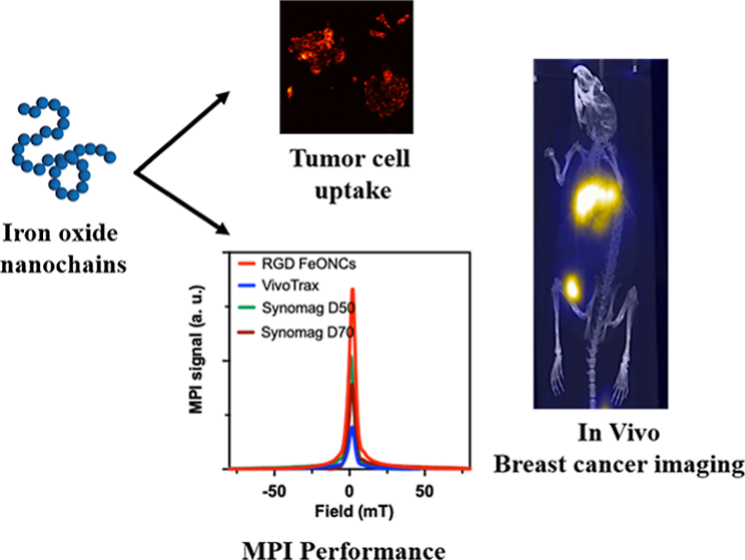

The advancement of imaging technologies plays a crucial role in improving the diagnosis and monitoring of diseases, including cancer. This study introduces a new design of iron oxide-based nanoparticles specifically developed for magnetic particle imaging (MPI), aimed at tracking and diagnosing breast cancer more effectively. By precisely controlling the size, shape, and magnetic properties of these nanoparticles, we enhance the responsiveness of MPI, resulting in an increased signal. In our research, we established a novel synthetic route for fabricating iron oxide nanochains (FeONCs) characterized by their uniform shape and size, which contribute to high magnetic properties suitable for MPI applications. Initial results indicate these FeONCs exhibit superior magnetic properties compared to conventional spherical superparamagnetic iron oxide nanoparticles, nanocubes, and reported nanoworm-type structures. Magnetic relaxometry studies revealed that FeONCs provide higher sensitivity than the commonly used VivoTrax Synomag D50 and D70 in MPI. Further, the size and shape of FeONCs significantly influence cellular uptake. In vivo experiments using orthotopic breast cancer mouse models allow us to assess the biocompatibility and magnetic characteristics of the nanoparticles, confirming their imaging efficacy. Furthermore, by conjugating these nanoparticles with the RGD peptide, we enhance their ability to specifically target breast cancer, establishing them as promising tracers for in vivo MPI applications characterized by high sensitivity. Thus, our findings highlight that FeONCs significantly improve imaging quality, facilitating the early detection and accurate monitoring of breast cancer. This paves the way for innovative diagnostic strategies and personalized treatment options. Future research will focus on fine-tuning the surface chemistry of these nanoparticles to further enhance the targeting efficiency and optimization of their practice in clinical applications, particularly for MPI-based hyperthermia therapy.

## 1 Introduction

Sophisticated imaging tools are necessary for breast cancer detection due to rising incidence rates and the complexity of breast cancer pathologies.^1−3^ Traditional modalities, such as mammography, while effective, have limitations, including a high rate of false positives, particularly in women with dense breast tissue.^4−6^ Additionally, mammography exposes patients to ionizing radiation, and conventional ultrasound can struggle with sensitivity and specificity.^7^ As a result, there is an urgent need for innovative imaging techniques that can enhance the diagnostic accuracy and improve early detection. Magnetic particle imaging (MPI) has emerged as a leading candidate for quantitative tomographic imaging tools to meet these challenges.^8−11^ MPI utilizes superparamagnetic iron oxide nanoparticles (SPIONs) to achieve high-resolution imaging with excellent signal properties.^12,13^ Unlike conventional imaging modalities such as magnetic resonance imaging (MRI), positron emission tomography (PET), X-ray computed tomography (CT), and ultrasound, MPI offers unique advantages in visualizing the distribution of nanoparticles within biological tissues, particularly in oncology applications. Based on tracer signal quantification, MPI can provide spatiotemporal resolutions that facilitate detailed representations of tumor vasculature and cellular activity, which sets it apart from other imaging methods.^8,14,15^

The performance of MPI is significantly influenced by the imaging tracer properties, particularly the shape and size of the iron oxide nanoparticles used as tracers. Optimizing these characteristics of iron oxide is crucial for enhancing the imaging sensitivity, biodistribution, and cellular uptake. The optimization of nanoparticle size can directly affect the imaging performance; for instance, in a study,^16^ specific size fractions of SPIONs (around 13.1 nm) obtained by sequential centrifugation methods provided optimal longitudinal and transverse relaxivity values, thereby improving the overall imaging capabilities compared to larger or smaller counterparts. This highlights the significance of precise size control in the design and application of iron oxide nanoparticles (FeONPs) for effective MPI use.

Smaller, uniform nanoparticles can penetrate tissues more effectively, allowing for improved targeting of tumor cells, leading to enhanced signal generation in that particular region.^17^ In addition to size, the shape of FeONPs significantly influences MPI performance.^18,19^ Particles engineered into specific shapes can exhibit different magnetic properties and interactions with external magnetic fields, which, in turn, can affect their detectability and the quality of the images produced.^20^ For instance, cubic iron oxide nanoparticles have shown promising results due to their unique characteristics, such as lower proportions of disordered surface spins, which contribute to larger saturation magnetization.^19^ The shape can also affect nanobio interaction, that is, how well nanoparticles can be distributed and tracked in vivo. Certain shapes are better suited for specific applications, such as improved cellular uptake or targeted delivery in imaging applications, which can enhance the resolution and accuracy of MPI.^19,21−23^ These size/shape-dependent properties not only affect the magnetic responsiveness of the nanoparticles but also determine their pharmacokinetics and biocompatibility, which are vital for successful clinical applications.^24,25^

The surface coating of FeONPs plays a critical role in determining their interactions with biological systems, particularly in terms of opsonization and the formation of a protein corona that develops when FeONPs encounter biological fluids such as blood serum. Surface coatings affect how nanoparticles are perceived by the immune system, determining their biodistribution, cellular uptake, and blood half-life.^26,27^ For instance, hydrophilic and neutral coatings, such as poly(ethylene glycol) (PEG), can minimize protein adsorption, thereby reducing opsonization and enhancing the circulation time of the nanoparticles within the bloodstream. Conversely, coatings that are hydrophobic or positively charged can promote stronger interactions with plasma proteins, leading to the rapid formation of a hard protein corona that facilitates opsonization and, consequently, faster clearance by phagocytic cells.^28,29^ Conjugation of FeONPs with RGD peptides can facilitate specific interactions with integrin receptors at tumor sites and promote the desired biological responses while also protecting against rapid immune clearance. Thus, RGD peptides significantly improve the pharmacokinetics and therapeutic delivery of FeONPs.^30,31^

Therefore, understanding the interplay between nanoparticle design, surface modifications, and imaging performance is essential for advancing MPI technology and its application in cancer diagnostics and treatment monitoring.

At present, the utilization of MPI for bioimaging and cell tracking primarily relies on established FeONPs such as VivoTrax/Resovist. Both have the same composition and material but are sold by different names for different applications. VivoTrax is used for MPI applications, and Resovist is for MRI applications. These commercially available agents were originally formulated for *T*_2_-weighted MRI, raising concerns about their suitable usages for MPI applications.^32,33^ Studies indicate that only a minor fraction, specifically less than 3%, of the large nanoclusters present in these formulations contribute to the MPI signal.^12^ In a notable advancement, Bulte et al. introduced a method that employed monodispersed superparamagnetic FeONPs, achieving enhanced MPI performance compared to Feridex and Resovist.^34^ Another commercially available tracer called Synomag-D (30, 50, 70) showed a good performance in terms of resolution and sensitivity compared to VivoTrax.^35−37^ However, several limitations hinder its utility for in vivo MPI applications, notably biocompatibility, dynamic range, and particle behavior within biological environments.^38^ In a study, Remmo et al. showed that the use of Synomag-D30 resulted in a large dynamic magnetic property under physiological conditions.^39^ The same group showed that Synomag-D50 showed a false-positive MPI signal since the particle gets aggregated in the biological conditions and detached from the cell surface.^40^

Overall, the current approaches to MPI reveal significant limitations tied to the inadequacies of existing magnetic tracers, even though MPI’s potential detection sensitivity and stable signal over time as compared to the radioactive decay process found in PET.^41^ Therefore, creating high-performance magnetic tracers with consistent sizing and a unique structure that aligns with MPI principles is essential for advancing the efficacy of MPI-based bioimaging and cell tracking methods.

Here, we report a new class of nanoparticle imaging agents for targeting breast cancer, validated using MPI. We have synthesized iron oxide nanochains (FeONCs) to enhance MPI signals for in vivo and in vitro applications. There are a few reports which highlight the synthesis of nanochained magnetic nanoparticles.^18,42−45^ Magnetic nanochains, typically formed through the alignment and aggregation of magnetic nanoparticles, can take several forms depending on the synthesis method and conditions. One common type is linear magnetic nanochains, constructed by aligning superparamagnetic nanoparticles along magnetic field lines.^42,45^ Another reported structure includes branched or tree-like magnetic nanochains, designed to enhance the magnetic moment for application in microfluidic devices.^43^ In other studies, such chain-type structures were prepared by coprecipitation synthetic methods followed by silica shell coating and magnetic field lines for the assembly.^44,46^ Although these nanochain-type structures have good magnetic properties, they have not been used for MPI applications due to complex design requirements, loss of individual superparamagnetism in chains, and challenges in achieving an optimal magnetic response for MPI. Their intricate structures can lead to convoluted magnetic signals, unintended aggregation, and difficulties in ensuring biocompatibility, which hinder large-scale clinical applications. Additionally, competition from alternative nanostructures, such as single-core SPIONs with well-established performance, further limits the practical adoption of magnetic nanochains for MPI. Specifically, in one of the reports, Colson et al. pointed out the formation of nanochain-type structures to improve the MPI response, but they have not used these structures for biological applications.^18^

Our magnetic susceptibility studies showed that the prepared FeONCs showed a magnetic moment that is four times higher than that of spherical iron oxide nanoparticles and 1.8 times higher than that of iron oxide nanocubes. The FeONCs were conjugated with DSPE-PEG or RGD peptides to improve the solubility and targeting ability. RGD-based FeONCs accumulated in the tumor earlier and with enhanced MPI signals compared to non-RGD and control spherical FeONPs. The study here highlights the importance of structure- and shape-related fabrication of FeONPs for better MPI responsiveness for breast cancer detection with targeting ability and can be used for MPI-guided hyperthermia applications shortly.

## 2 Results

### 2.1 Synthesis and Characterization

**Scheme 1:**
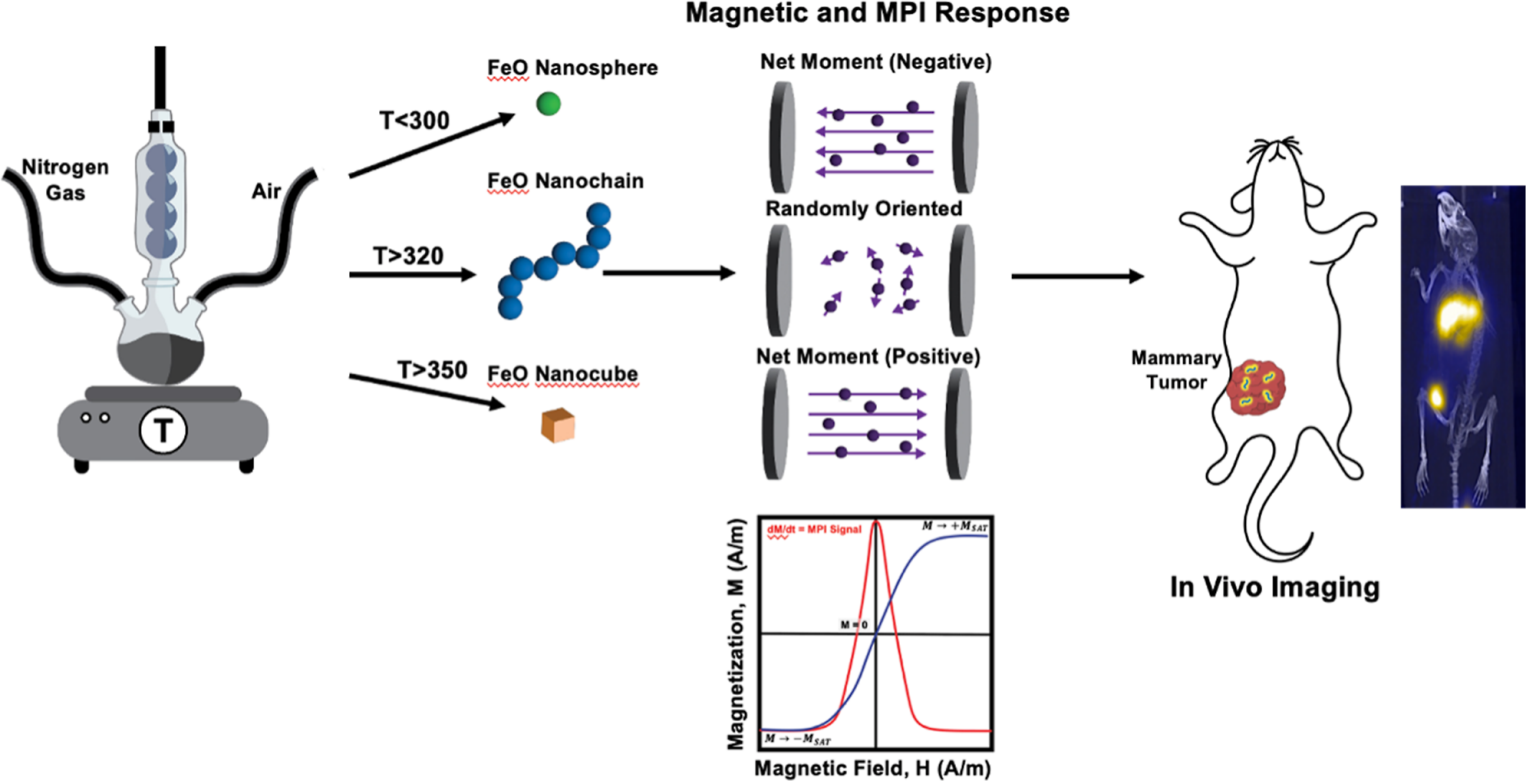
Schematic Representation for the Synthesis, Magnetic Properties, and MPI Imaging Application of FeONCs

Scheme 1 represents the synthetic route and the working principle of FeONCs as an MPI tracer. The iron oxide nanochain (FeONC) was synthesized using a thermal decomposition (for details, see the Supporting Information).^47,48^ Briefly, first, we prepared an iron–oleate complex as a precursor to fabricate FeONP and formed FeONCs. The iron–oleate complex is mixed with oleic acid and 1-octadecene and subjected to thermal decomposition at 320 °C under an inert environment. Variations in the temperature resulted in spherical iron oxide NPs (FeONPs) (*T* < 320 °C) or cubic-shaped iron oxide NPs (FeONCB) (*T* > 340 °C). Controlling the amount of octadecene is important since it acts as a high-boiling solvent during the thermal decomposition of the iron–oleate complex, and oleic acid acts as a surfactant, coating the growing nanoparticles to prevent agglomeration and controlling their size and shape.

**Figure 1:**
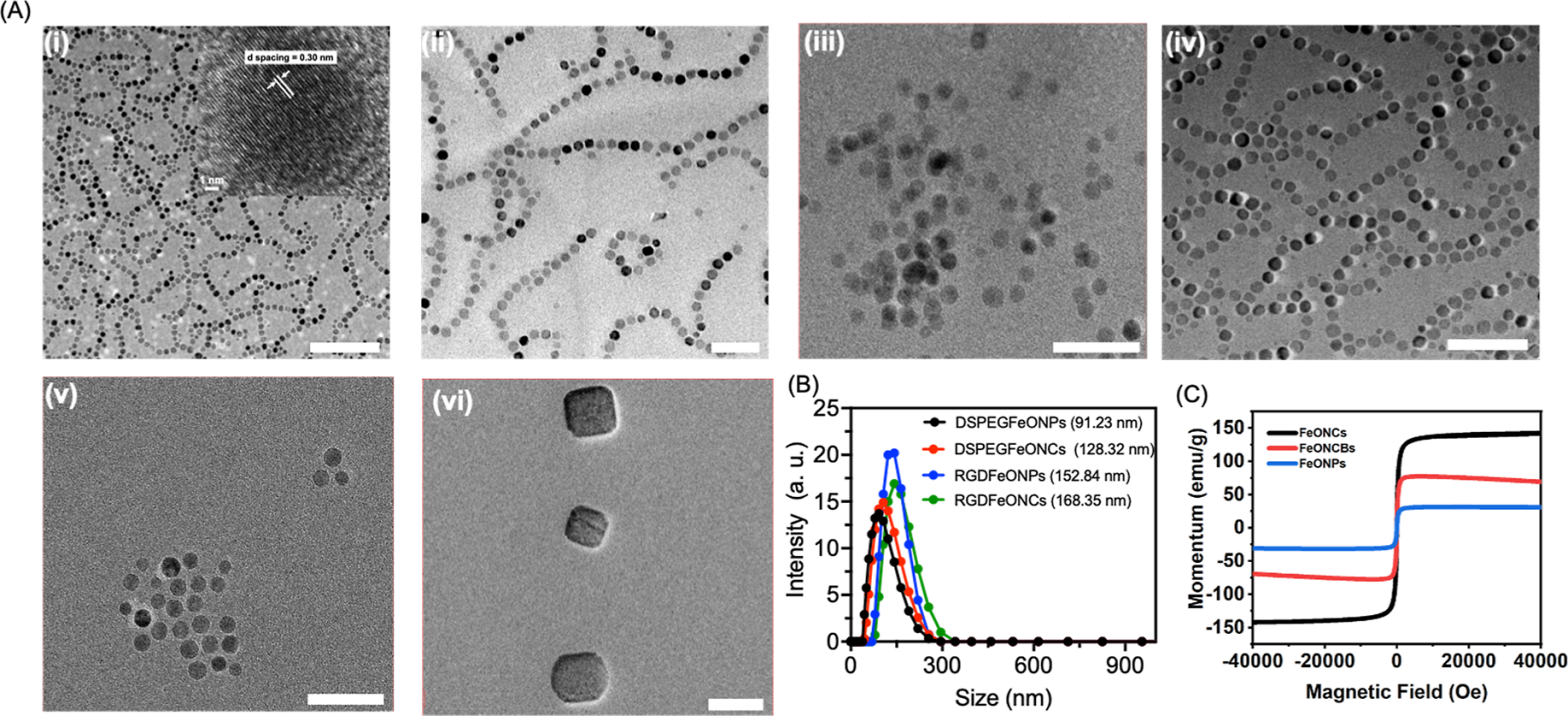
Characterization of prepared iron oxide nanochains and nanoparticles. (A) TEM images for (i) FeONCs, (ii) DSPE-PEG FeONCs, (iii) DSPE-PEG FeONPs, (iv) RGD FeONCs, (v) RGD FeONPs, and (vi) FeONCB. Scale bar = 100 nm. The inset in figure (i) shows the high-resolution TEM images for FeONCs, with the *d* spacing value of 0.30 nm, which corresponds to the (220) plane. Scale bar = 1 nm. (B) Dynamic light scattering experiments for prepared nanochains and nanoparticles using DSPE-PEG and RGD surface modifications. The concentration of Fe is made to 50 μg/mL for the DLS measurements. (C) SQUID magnetometry measurements for the prepared FeONCs, FeONCBs, and FeONPs at room temperature. Magnetization curves were obtained from −50,000 to 50,000 (Oe).

The prepared FeONCs were characterized by transmission electron microscopy (TEM) (Figure 1A(i)). TEM images showed a nanochain structure composed of spherical iron oxide nanoparticles with an average diameter of 17.4 ± 2.1 nm. The high-resolution TEM studies showed that FeONCs showed a magnetite crystal lattice with an interplanar distance *d* spacing of 0.264 and 0.301 nm corresponding to the (311) and (220) planes of the crystalline Fe_3_O_4_ (inset Figure 1A(i)). To improve the water solubility and biocompatibility, the surfaces of FeONCs were modified with DSPE-PEG. The ζ potential of PEG-FeONC was found to be −10.8 mV, with an average hydrodynamic diameter of 128.2 ± 3.3 nm (Figures 1B and S1). TEM images showed that PEGylation does not alter the nanochain structures but improves the water solubility and stability (Figure 1A(ii)). To study in detail the effect of FeONCs as MPI contrast agents, we have used spherical iron oxide nanoparticles (FeONPs) as a control (from Sigma-Aldrich). The FeONPs have an average size of 15 nm, as confirmed by TEM, and were modified with DSPE-PEG for water solubility and stability (Figure 1A(iii)). The FeONPs showed a ζ potential of −13 ± 3 mV with a hydrodynamic diameter of 91.23 ± 5.1 nm (Figure 1B and S1).

To improve the targeting ability of prepared nanochains and nanoparticles, we modified the surface of nanoparticles with RGD peptide using the EDC cross-linker as described in the Supporting Information. The conjugation of DSPE-PEG and RGD to the NPs was confirmed by Fourier transform infrared spectroscopy (FT-IR). FT-IR showed the characteristic stretching vibrations for the Fe–O– bond at 563 cm^–1^, and the ester and amide bonds from DSPE-PEG and RGD peptide (1730 (–C=O ester), 1642 (amide –C=O I and 1550) (amide C=O (II) cm^–1^). The stretching vibrations at 1103 cm^–1^ indicated the presence of long –C–O– ether linkages from the PEG molecule on the surface of FeONCs and FeONPs (Figure S2). As shown in Figure 1B, the hydrodynamic size and zeta potential of NPs are similar after conjugation with RGD (Figure S1). The TEM showed that after the surface modification with RGD, there is no change in the morphology of FeONCs and FeONPs, indicating they are stable and there is no aggregation (Figure 1A(iv,v)).

### 2.2 Magnetic Moment Measurement

MPI operates by detecting the unique magnetic signatures of these nanoparticles when subjected to an external magnetic field. The technology utilizes magnetic field gradients to focus on specific regions, which is called the field-free point (FFP), which encodes the spatial localization of SPIONs in the body. MPI images are produced as the FFP rapidly sifts, which causes a change in the magnetization of the SPIONs within the FFP. The superparamagnetic properties of the nanoparticles are crucial for MPI’s operation. These properties allow the nanoparticles to be magnetized rapidly in response to the external magnetic field.^49^ Only when the SPIONS are within the FFR can they respond to the external magnetic field, which is seen as magnetization, and can be transformed into the images we can visualize. Recently, Wang et al. reported that cubic-shaped FeONPs showed better magnetization and MPI properties than spherical FeONPs due to the smaller surface anisotropies and less disordered spins in their lattice, which showed a higher magnetization saturation (*M*_s_) and magnetic susceptibility (χ).^19^ Our as-prepared FeONCs showed *M*_s_ and χ, which are 4 times higher than spherical FeONPs, which we purchased from Sigma-Aldrich with an average diameter of 15 nm. The potential magnetic hysteresis of the nanoparticles was measured by the superconducting quantum interference device (SQUID) magnetometer at 300 K. MH curves for the samples are presented in Figure 1C. The saturation magnetization was normalized by using iron mass in the sample quantified by inductively coupled plasma optical emission spectroscopy (ICP-OES) optical emission spectroscopy (ICP–OES). As shown in Figure 1C, the magnetic measurement studies showed a superparamagnetic nature for the FeONCs. This indicates they showed magnetic properties only under an external magnetic field, which is one of the prerequisites for MPI tracers. The saturation magnetization was observed at 144.34 emu/g for FeONCs, whereas the magnetic saturation for FeONP was observed at 33.56 emu/g. We further studied the magnetic properties of iron oxide nanocubes (FeONCB), which we prepared by varying the temperature conditions, as shown in Scheme 1. The iron oxide nanocubes showed a magnetic moment that is 1.8 times less than the FeONC with a saturation magnetization of 68.28 emu/g and matched well with one of the previous reports.^19^ The high magnetization value for iron oxide chain NPs is reported previously depending on the particle size and iron core; thus, the obtained value of magnetization for FeONCs is acceptable.^50,51^ In one of the reports, Park et al. reported the synthesis of iron oxide nanoworms having an average diameter of 50–80 nm.^52^ This nanoworm-type structure showed a magnetic moment of 74.2 emu/g compared to the spherical FeONP counterparts, which showed a magnetic saturation of 53.5 emu/g. The authors postulated that the elongated structure of the nanoworm apparently enhances the orientation of the magnetic moments of the individual nanoparticle constituents, increasing the net magnetization and an improved MRI signal for tumor detection. Thus, the prepared FeONCs with a more elongated structure than nanoworms will show an enhanced magnetization property, as observed by the SQUID measurements. Thus, FeONCs will possess a large magnetic volume under an applied magnetic field. This increased magnetic moment enhances their ability to respond to magnetic fields quickly and effectively, making them ideal candidates for MPI applications. Moreover, the anisotropic shape of FeONCs influences their magnetic properties, leading to greater directional dependence in the magnetic response compared with the isotropic nature of spherical or cubic shapes. Due to the high hydrodynamic volume of FeONCs, they possess lesser Brownian movements, leading to stable magnetic field orientations and higher MPI signal enhancement.^36^ Similarly, the self-assembled FeONCs structure improves the Neels temperature threshold, allowing higher magnetic ordering for enhanced contrast for MPI applications.^53^

### 2.3 Cell Viability and Cellular Uptake

**Figure 2:**
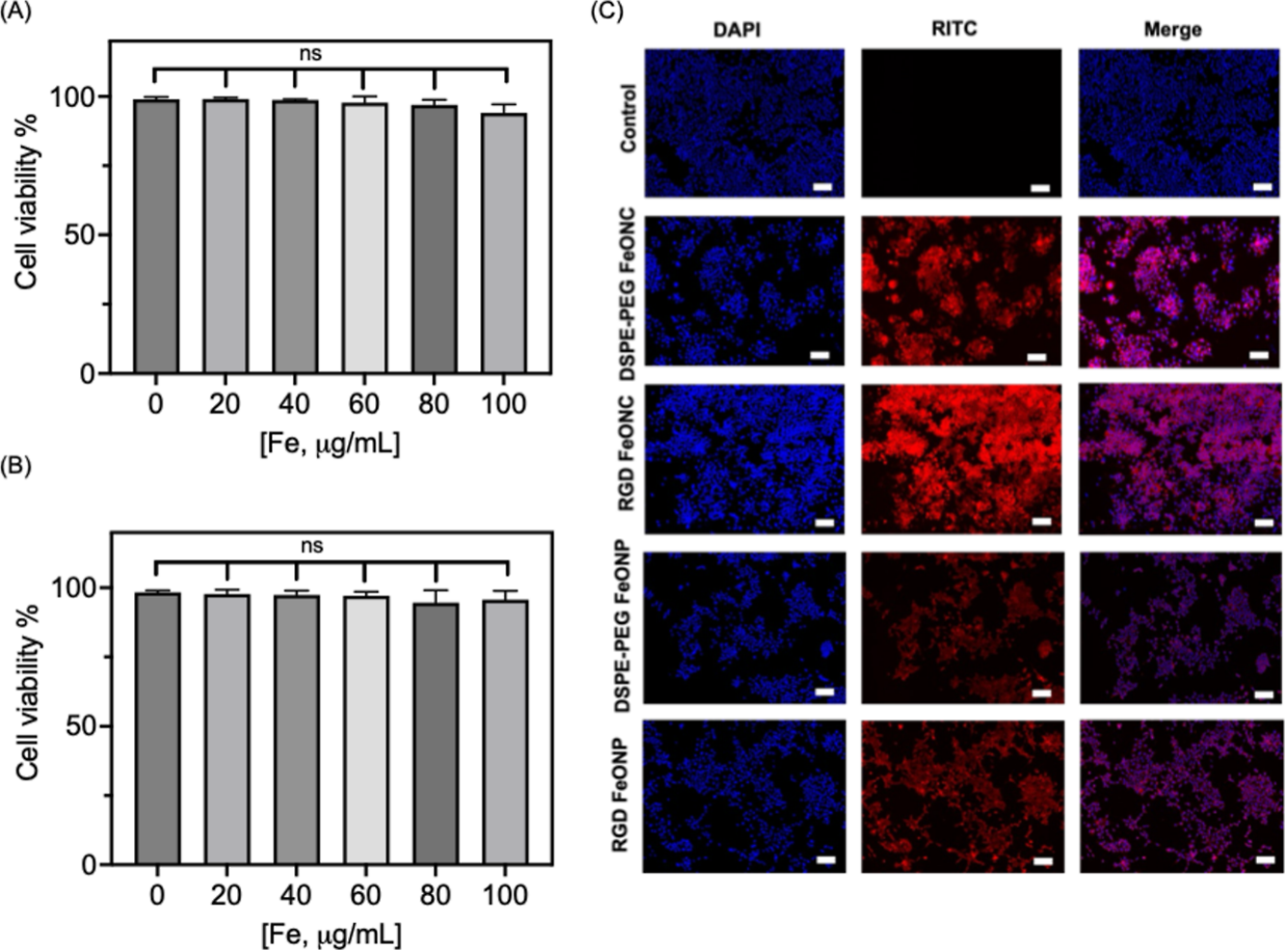
Cell viability and cellular uptake of NPs. (A,B) MTT assay for DSPE-PEG FeONCs and RGD FeONCs using 4T1 breast cancer cells with varying concentrations of [Fe]. (C) Fluorescence microscopic images for nontreated control, DSPE-PEG/RGD-FeONC and DSPE-PEG/RGD-FeONPs. Blue = DAPI, RITC = NPs. The concentration of Fe used is 25 μg/mL for the cellular uptake studies. Scale bar = 50 μm.

The prepared FeONCs modified with DSPE-PEG and RGD were studied for their biocompatibility using 4T1 breast cancer cells and mesenchymal stem cells (MSCs) (Figures 2A,B and S3A,B). Before this, the amount of Fe in the nanoparticles was measured by using inductively coupled plasma. The nanoparticles showed no toxicity using concentrations of FeONCs ([Fe] = 0 to 100 μg/mL, *n* = 5) with DSPE-PEG and RGD on their surfaces (Figure 2A,B).

For the cellular uptake studies, the nanoparticles were conjugated with a fluorescent marker, rhodamine B isothiocyanate (RITC) (Supporting Information for synthetic details). The DLS studies showed a slight increase in the size of the NPs after conjugation with RITC to the particle backbone (Figure S4A). Extinction and emission spectra for the NPs showed the characteristic absorption and emission peaks for RITC at 560 and 590 nm, respectively, confirming the conjugation of RITC to FeONCs and FeONPs (Figure S4B,C). FT-IR studies showed the characteristic vibrational peaks for RITC at 1700 cm^–1^ (–COOH), 750 cm^–1^ (–C=S), and 1088 cm^–1^ (–C–S–) and for DSPE-PEG at 1710 cm^–1^ (–C=O), 1108 cm^–1^ (–C–O–C), and 2842 cm^–1^ (–CH_2_–) confirming the presence of RITC and DSPE-PEG on FeONC and FeONPs. The vibrational frequencies for the Fe–O bond were obtained at 536 cm^–1^, further supporting the conjugation of RITC-DSPE-PEG to FeONC and FeONP (Figure S4D). As shown in Figure 2C, the nanoparticle uptake is confirmed by the RITC signals in the NP-treated cell lines as compared to the nontreated cells. The RITC uptake was more prominent using RGD FeONC than the other nanoparticles. As compared with FeONCs, the cellular uptake of FeONPs is less prominent. The physicochemical properties of nanomaterials significantly influence their interaction with cells. Factors such as size, shape, surface charge, and hydrophobicity play a vital role in cellular uptake. Various endocytotic pathways, such as clathrin-mediated and caveolae-mediated endocytosis, are involved in different sizes of nanoparticles.^54,55^ Clathrin-mediated endocytosis mainly governs submicrometer particles of 100–350 nm and is crucial for cellular uptake, as blocking it decreases nanoparticle internalization. Caveolae-mediated endocytosis, on the other hand, is responsible for the uptake of nanoparticles within the 20–100 nm range. This intricate interplay underscores the complexity of nanomaterial–cell interactions. Since there is not much difference in the size of FeONCs and FeONPs cores, the higher internalization of FeONCs in the cancer cells is due to the stronger adhering nature of the nanochain assembly based on multivalent interactions on the cell membrane than the spherical FeONPs. The spherical FeONPs provide fewer binding sites to interact with the cell membrane because of their curved surface and show relatively less internalization.^54,56^

### 2.4 MPI-Based Relaxometry

The performance of MPI is critically dependent on the magnetic behavior of SPIONs, which can be characterized by magnetic relaxometry. These studies provide insights into the dynamics of SPION magnetization under an oscillating magnetic field and are crucial for evaluating and optimizing sensitivity and resolution.^57,58^ The technique involves turning off the selection field and applying negative and then positive magnetic fields, transitioning the SPIONs between a negative magnetic saturation and a positive state. The measurement yields the derivative of the Langevin function known as the point spread function (PSF). The PSF height indicates the peak signal intensity of the SPION, while the full width at half-maximum (fwhm) reveals spatial resolution; a narrower response reflects better resolution, and a higher peak signal intensity per iron mass indicates improved sensitivity. These parameters are essential for enhancing SPION performance in imaging and targeted drug delivery applications.

**Figure 3:**
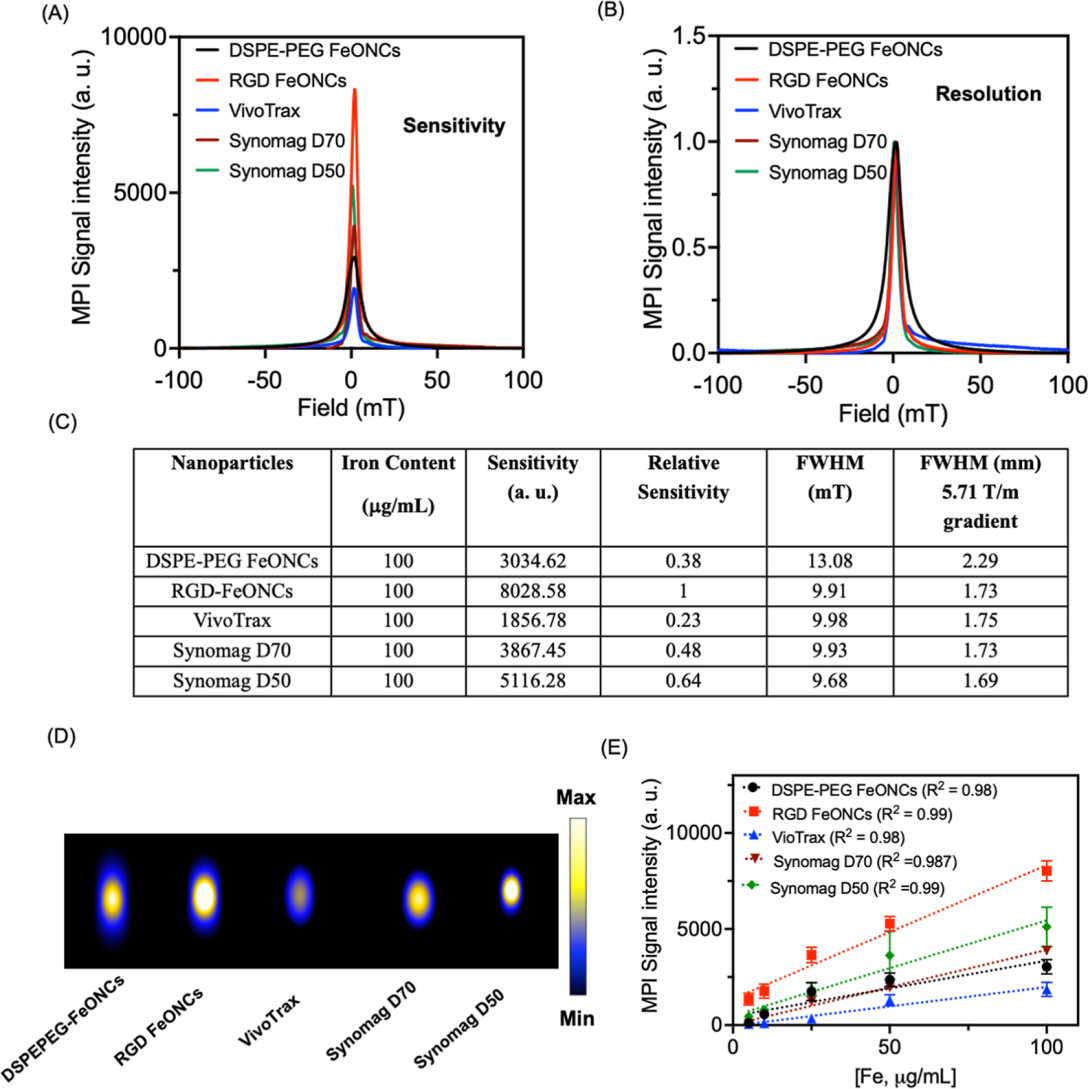
Comparison of sensitivity and spatial resolution of NPs. (A) PSF for DSPE-PEG FeONCs, RGD FeONCs, VivoTrax, Synomag D70, and Synomag D50. RGD FeONCs showed higher signal sensitivity than all other tracers studied for the same iron content [Fe, 100 μg/mL]. (B) Normalized signal resolution for DSPE-PEG FeONCs, RGD FeONCs, VivoTrax, Synomag D70, and Synomag D50. (C) Table to represent the sensitivity and resolution of each nanoparticle for the same concentration of iron [Fe, 100 μg/mL]. (D) 2D MPI images for DSPE-PEG FeONCs, RGD FeONCs, VivoTrax, Synomag D70, and Synomag D50 for 100 μg/mL iron content. (E) Linear correlation for the MPI signal intensity with varying iron content for the NPs and commercial tracers studied.

Various concentrations (5, 10, 25, 50, and 100 μg/mL) of DSPE-PEG/RGD-FeONCs, VivoTrax, Synomag D70, and Synomag D50 were prepared and tested for relaxometry studies (Figure 3A–E). As shown in Figure 3A, both DSPE-PEG and RGD FeONCs had a narrower PSF with a high signal intensity per iron mass, indicating improved tracer characteristics for MPI than VivoTrax. The DSPE-PEG FeONCs showed a comparable signal intensity with Synomag-D70 and a lower signal intensity than Synomag-D50. The RGD FeONCs showed improvements in signal intensity, which is higher than those of the commercially available tracers (4.3 times higher than VivoTrax, 2.03 times higher than Synomag-D-70, and 1.56 times higher than Synomag-D50) (Figure 3A–C). The fwhm calculation after normalization showed that RGD FeONCs showed a resolution comparable with that of the commercially available tracers such as VivoTrax and Synomag-D70 but a slightly lower value than that of Synomag-D50 (Figure 3C). Meanwhile, DSPE-PEG FeONCs showed a lower resolution than commercially available tracers and RGD FeONCs. This indicates that RGD not only allows targeting ability toward tumors but also helps assemble the FeONCs in a solution for high magnetization and magnetic volume. The corresponding MPI images show a similar trend, as shown in Figure 3D, indicating that RGD FeONCs contribute to higher MPI signal intensity when compared to other tracers studied. All tracers demonstrated a linear correlation between the signal intensity and Fe concentration, as shown in Figure 3E.

### 2.5 FeONC as the MPI Contrast Agent

**Figure 4:**
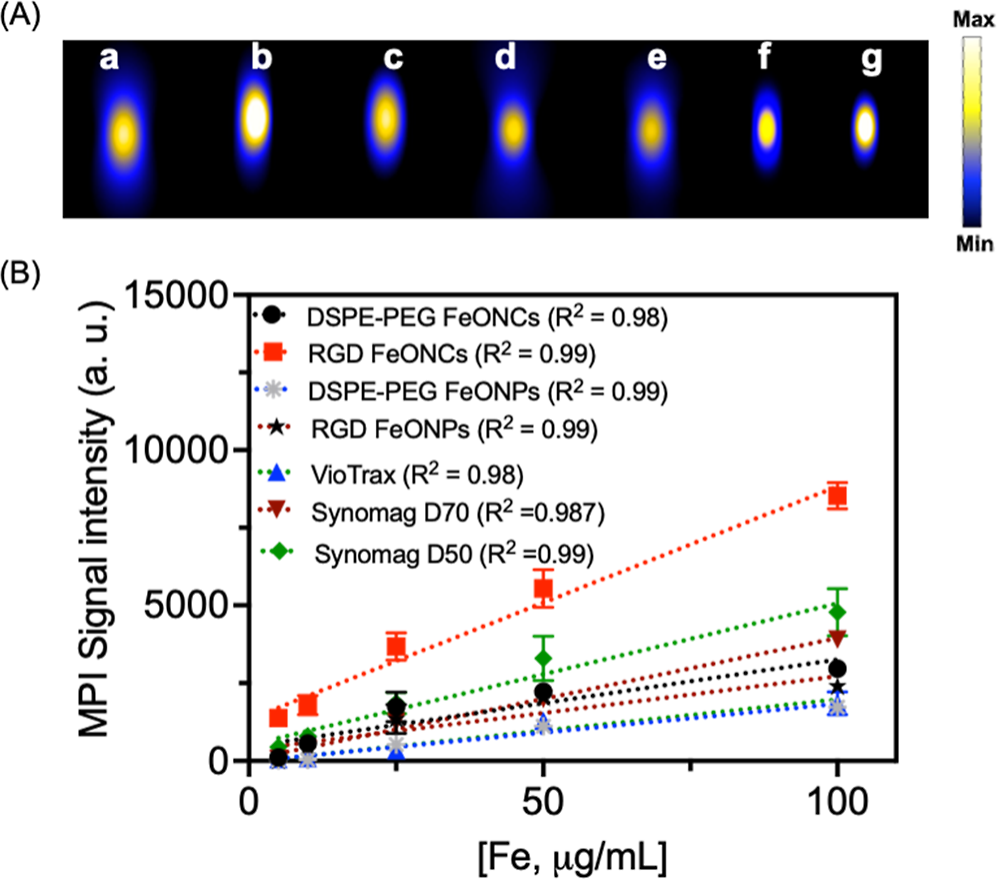
2D MPI images for various NPs used for the study. 2D MPI images for various NPs and commercial tracers used for the study. (A) 2D MPI images for (a) DSPE-PEG FeONCs, (b) RGD FeONCs, (c) DSPE-PEG FeONP, (d) RGD FeONP, (e) VivoTrax, (f) Synomag-D70, and (g) Synomag-D50, respectively. The concentration of Fe is 100 μg/mL. (B) Linear correlation curve for the MPI signal intensity with varying [Fe] content in different nanoparticles studied.

After physicochemical characterization and in vitro studies, the prepared NPs were investigated for their performance as MPI tracers in vitro. MPI was utilized to visualize and quantify the increasing amounts of FeONC and FeONP in aqueous solutions within test tubes to determine sensitivity to iron content. To assess the performance of FeONCs in MPI, a head-to-head comparison was conducted using commercially available MPI tracers such as VivoTrax, Synomag-D70, and Synomag-D50. The 2D images for all these tracers for various Fe contents (5, 10, 25, 50, and 100 μg/mL, 100 μL volume) are represented in Figures 4A and in S5A–G. The MPI signals of all iron particles exhibited a linear relationship with the Fe amount, as depicted in Figure 4B. As Figure 4B illustrates, the RGD FeONCs showed a significant increase in signal intensity as compared with all tracers. RGD FeONCs showed a signal intensity that is 4.3 times higher than VivoTrax and 2.0 times higher than Synomag-D70 (Figure 4A). However, the signal intensity is 1.56 times higher than that of Synomag-D50, which has been widely used recently for MPI-based applications (Figure 2A). Compared to DSPE-PEG FeONPs and RGD FeONPs, the RGD FeONCs showed a signal enhancement which is 3.8 times higher with a good sensitivity (Figure 4A). As Figure S5A–G shows, the FeONPs-based tracers lose their sensitivity in lower concentrations beyond 25 μg. This shows that RGD peptide not only helps the tumor-targeting ability but also acts as a template for the assembly of NPs to enhance their MPI responsiveness by increasing the average magnetic volume for MPI imaging. The RGD FeONCs showed enhanced MPI signal compared to FeONP and VivoTrax at low concentrations, indicating the ability of FeONCs as an intense MPI tracer.

### 2.6 Differential Particle Uptake by Cell Types Using MPI

MPI enables precise detection of magnetic nanoparticle signals free from interference by surrounding tissues. Studies confirm its utility in tracking SPION-labeled cells for noninvasive, repeated imaging of transplanted cells, potentially applicable in clinical settings.^57,59−61^ We have studied three cell types—4T1 breast cancer, J774 macrophages, and MSCs—with FeONCs internalized through distinct pathways. Experiments were performed with a fixed Fe concentration of 100 μg/mL, with varying cell numbers. After 24 h, MPI analysis showed signal intensity correlated linearly with cell count, with J774 cells displaying three times stronger signals than MSCs and 4T1 cells (Figure S6A–C). J774 and MSCs internalize FeONCs primarily via endocytosis, which is influenced by particle size, shape, and surface properties. Smaller particles are internalized through receptor-mediated pathways, while macrophages exhibit robust endocytosis and prefer larger particles.^62,63^ Tumor cells, with limited uptake mechanisms, show lower nanoparticle internalization.^64,65^ The FeONC nanochains were quantified using MPI calibration curves and validated by ICP–OES after cell lysis (Figure S7).

### 2.7 In Vivo Tumor Targeted Imaging by MPI

**Figure 5:**
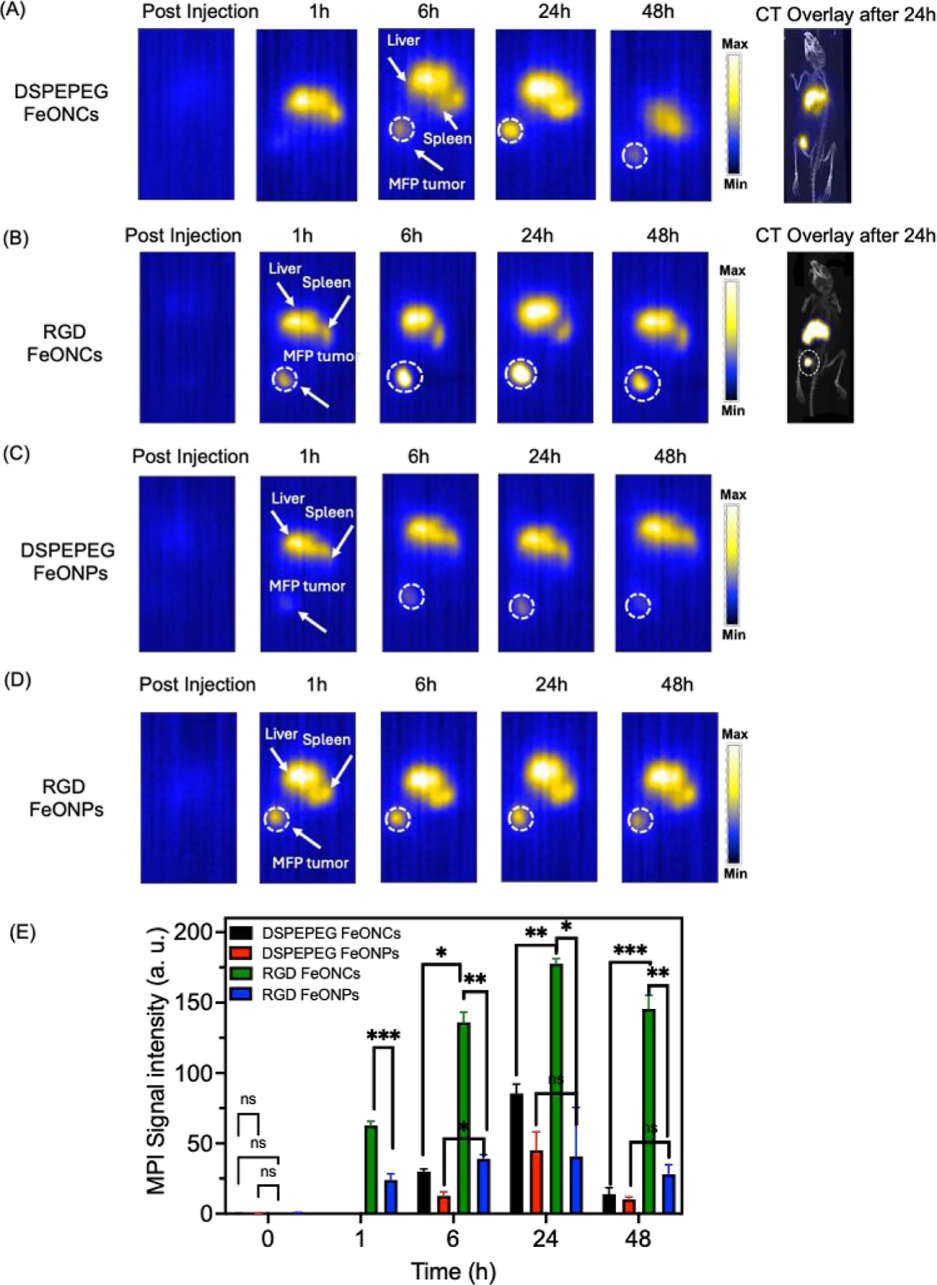
In vivo 3D MPI images for the orthotopic breast cancer mice model. 3D MPI images for (A) DSPE-PEG FeONCs, (B) RGD FeONCs, (C) DSPE-PEG FeONPs, and (D) RGD FeONPs using orthotopic 4T1 mammary fat pad (MFP) mice. A-B show the CT overlay images with MPI for DSPE-PEG and RGD FeONCs after 24 h of particle injection. (E) Quantitative measurement for the MPI signals in the tumor regions for various NPs studied in the breast cancer model, where *n* = 3, *P < 0.05, **P < 0.01, ***P < 0.001.

To evaluate the ability of the nanoparticles to target tumors and subsequent MPI detection, NPs with DSPE-PEG and RGD (100 μg/mL, 100 μL) were intravenously injected into mice bearing 4T1 breast tumors. These mice were imaged using 3D MPI immediately post NP injection, followed by imaging at 1, 6, 24, and 48 h post NP injection (Figure 5A–D). There was no apparent MPI signal immediately following NP injection due to the intravenous distribution of Fe, whereas after 1 h, all mice showed signals in the liver and spleen. Mice that were administered RGD NPs had MPI signals in the tumor at 1 h postinjection, with the RGD FeONC-injected mice showing stronger tumor signals (Figure 5B) versus mice injected with the RGD FeONP (Figure 5D). At 6 h postinjection, the DSPE-PEG FeONC- and DSPE-PEG FeONP-injected mice began to have quantifiable MPI signal; however, it was less than that of RGD-based NPs (Figure 5A,C). MPI demonstrated that the RGD FeONC and RGD FeONP accumulation peaked in the tumor site 24 h postinjection, with signals also remaining in the liver and spleen. This study shows that the RGD-based FeONC can accumulate in the tumor region and at an earlier time point compared to the non-RGD-based NPs. RGD FeONC showed a signal intensity that is 2.8 times higher than the RGD FeONP (Figure 5E), whereas the DSPE-PEG FeONCs showed a signal intensity that is 1.5 times higher than DSPE-PEG FeONPs (Figure 5E). 3D MPI images were coregistered with CT images after 24 h of NP injection to provide anatomical information. It clearly showed the accumulation of RGD FeONCs and DSPE-PEG FeONCs at the tumor site, as well as the liver and spleen (Figure 5A,B).

The uptake of nanoparticles by cells is a complex process influenced by several factors, including size, shape, surface charge, and the presence of surface modifications. Some of the previous studies show that anisotropic nanoparticles preferentially accumulate in cancer cells, contrasting with the behavior of spherical particles.^52,66^ The elongated geometry of nanochains facilitates a larger surface area for interactions with cell receptors and enhances their ability to navigate through the extracellular matrix, which is dense in tumors. The PEGylation improves the blood circulation time and the biocompatibility of NPs and can accumulate in tumors passively, whereas the RGD-based NPs show a targeted tumor accumulation.

Spherical FeONPs, even when PEGylated to improve biocompatibility and circulation time, may experience hindered uptake due to their shape. PEGylation effectively reduces the opsonization of nanoparticles, whereby recognition by the immune system occurs but does not significantly enhance the cellular binding affinity of the spherical particles. Thus, while spherical FeONPs can escape immune detection for a time, their overall uptake remains lower due to their geometric limitations.^67^ The elongated, anisotropic nanoparticles, such as nanoworms and nanochains, exhibit specific advantages; they tumble and rotate in blood flow, allowing them greater mobility toward the vessel wall and enhanced margination compared to spherical counterparts. This dynamic behavior not only facilitates proximity to endothelial cells but also influences their subsequent uptake into tumor sites, resulting in higher accumulation.

### 2.8 Ex Vivo Analysis of Tissues

**Figure 6:**
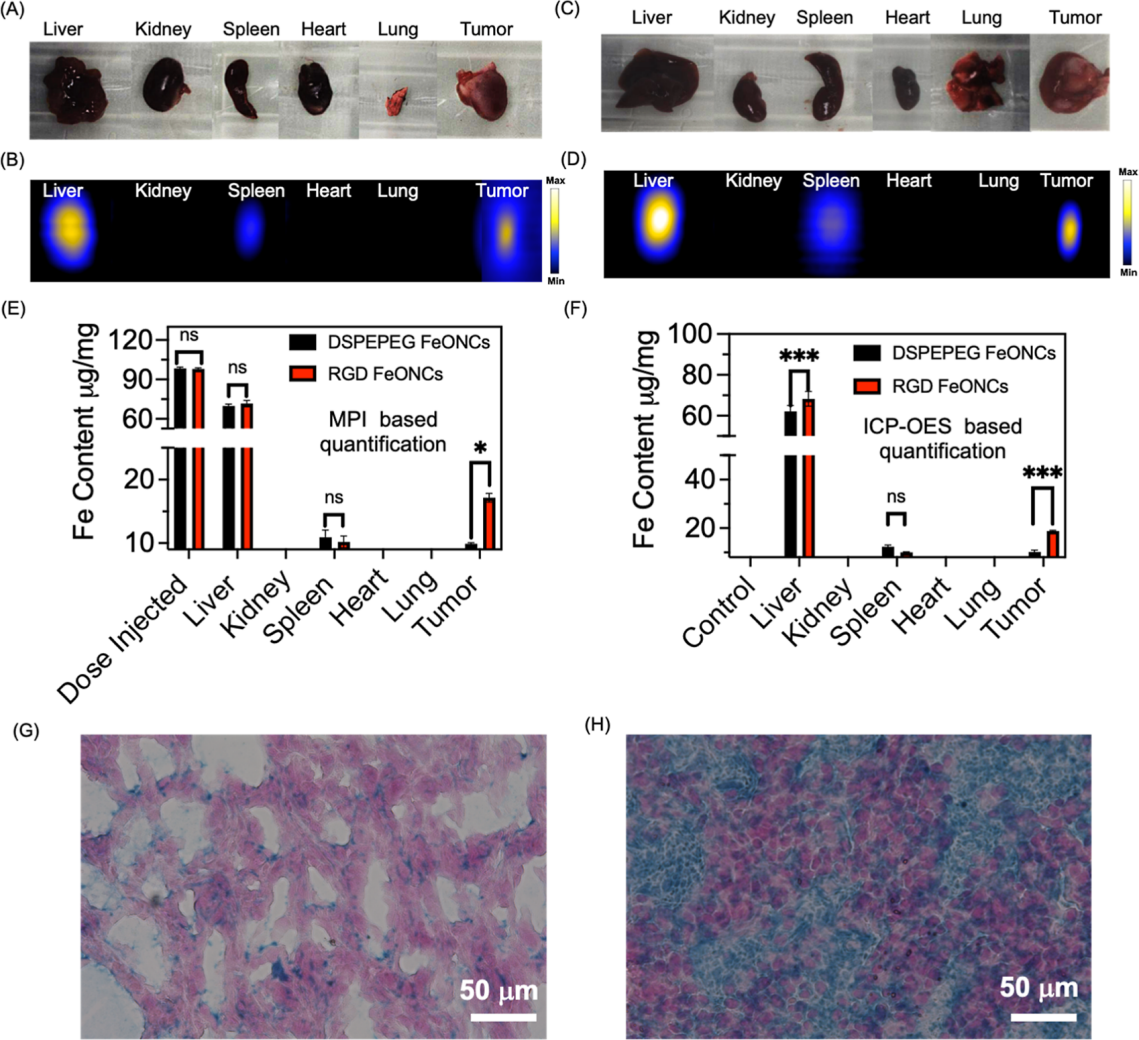
Ex vivo analysis of DSPE-PEG FeONCs and RGD FeONCs using MPI. (A) Digital photographs and (B) 2D MPI images showing the excised organs from mice injected with DSPE-PEG FeONCs [Fe, 100 μg/mL]. (C) Digital photographs and (D) 2D MPI images showing the excised organs from mice injected with RGD FeONCs [Fe, 100 μg/mL]. The mice are sacrificed after 24 h of injection of NPs through I V injection. (E,F) Quantification of Fe in the main organs of mice after DSPE-PEG and RGD FeONCs injection using MPI and ICP–OES, respectively (*n* = 3). Prussian blue stained (iron showed blue) followed by nuclear fast red counterstain for (G) DSPE-PEG FeONC and (H) RGD FeONCs, respectively. Scale bar 100 μm. All the data were analyzed by *t*-test using Graph Pad (***p ≤ 0.001; *p ≤ 0.01).

To validate the results obtained from in vivo imaging, mice were euthanized 48 h after the injection of nanoparticles, followed by the extraction and imaging of organs via MPI. The biodistribution of nanoparticles, both those engineered with RGD sequences and those not with RGD conjugation, was assessed by quantifying MPI images acquired from the excised organs (Figure 6A–D). The signal quantification revealed robust MPI signals in the tumor as well as in the liver and spleen (Figure 6B,D). Using the MPI signal calibration curve as in Figure 4E, the iron content in the excised organs and tumors was calculated. As shown in Figure 6E, a large uptake of Fe was found in the liver for both DSPE-PEG and RGD-based FeONCs (∼68 μg/mg from the injection dose of 100 μg/g). The MPI signal quantification in the tumor showed an uptake of 17.2 μg/mg of Fe for the RGD FeONCs, whereas 9.2 μg/mg of Fe in the tumor was observed for the non-RGD FeONCs-treated mice (Figure 6E). Thus, the RGD-based FeONCs showed 17.2% uptake of Fe in the tumor, whereas non-RGD FeONCs showed an uptake of 9.2%. An uptake of ∼10–11 μg/mg was found in the spleen for the mice, which are treated with either DSPE-PEG or RGD FeONCs. To validate this further, we have performed ICP–OES measurements with the excised organs and tumors for Fe quantification, which offer a sensitive and accurate determination. As shown in Figure 6F, ICP–OES studies showed a statistically significant value for the uptake of Fe in the tumor treated with RGD FeONCs compared to the DSPE-PEG FeONCs. Thus, both MPI and ICP–OES-based quantification showed an uptake of Fe in the RGD FeONCs, which is 1.8 times higher than in DSPE-PEG FeONCs-treated mice. In an additional step to confirm the accumulation of FeONCs in the tumors, the extracted tissues were subjected to examination using Prussian blue staining (Figure 6F,G). The substantial blue coloration observed in these sections provided strong evidence for the presence of both non-RGD- and RGD-functionalized FeONCs within the tumor tissues.

## 3 Discussion

The relentless pursuit of improved imaging technologies has become paramount in the ongoing battle against breast cancer. The unique features of iron oxide-based nanoparticles, particularly in the context of MPI, offer promising avenues for enhancing diagnostic accuracy and monitoring effectiveness. However, optimizing the size, shape, and magnetic properties of FeONPs is still challenging due to complicated synthesis, controlling aggregation, and limited systemic targeting abilities toward tumors. Here, we introduce the synthesis of iron oxide nanochains (FeONCs) that represent a noteworthy advancement in nanoparticle engineering. By precisely controlling their size and shape, we have enhanced both the responsiveness of MPI and its spatial resolution for imaging. The nanochain assembly enhanced their magnetic volume and anisotropic factors, as SQUID measurements confirmed it and showed a magnetic susceptibility that is 4 times higher than spherical NPs. The lack of a magnetic hysteresis loop indicated that these FeONC possess a superparamagnetic nature suitable for MPI applications. Initial magnetic relaxometry studies indicate that these nanoparticles provide elevated sensitivity and demonstrate reduced background noise during analyses, which is crucial for distinguishing subtle differences in tissue characteristics. Such improvements have critical implications for the early detection and accurate monitoring of breast cancer. Moreover, the impact of the structural parameters of FeONCs on their cellular uptake cannot be overstated. The distinct morphology of these nanoparticles plays a vital role in their interaction with different cell types, allowing for the more effective targeting of cancerous cells. By integrating this understanding into their nanoparticle design, researchers can potentially differentiate between various cell lines based on MPI signal intensity, which may lead to more personalized treatment strategies tailored to the biological characteristics of individual tumors.

In vivo experiments involving FeONCs have reaffirmed their potential as effective imaging agents in cancer medicine, showcasing both their biocompatibility and favorable magnetic characteristics.^68^ The importance of these properties lies not only in their ability to provide clear imaging but also in their capacity to facilitate the effective tracking of tumor responses to treatment. High sensitivity and spatial resolution make FeONCs exemplary agents for monitoring tumor dynamics, a necessity in modern oncology practices. The significant advancement in imaging quality provided by these nanoparticles sets a new standard for diagnostic techniques, ensuring that clinicians can identify pathological changes at earlier stages when they are more amenable to treatment.

Incorporating RGD peptides to enhance the targeting ability of FeONCs further exemplifies the innovative strides being made in this field. RGD peptides, known for their affinity to integrins overexpressed on tumor cells, can significantly improve the specificity of drug delivery systems, thus minimizing side effects associated with traditional therapies.^69,70^ This targeted approach, coupled with the advanced imaging capabilities of FeONCs, paves the way for the development of personalized therapeutic regimens that are both practical and patient-friendly.

As this research continues to unfold, future studies must prioritize refining the surface chemistry of the FeONCs. These modifications will be essential for maximizing targeting efficiency and optimizing their integration into clinical practice. Additionally, pursuing MPI-based hyperthermia represents a critical frontier in cancer treatment; harnessing the thermal properties of iron oxide nanoparticles during imaging sessions may enhance destructive effects on tumors while monitoring these changes in imaging. This dual capability of imaging and therapy exemplifies the potential of FeONCs as precision theranostics in transforming cancer management paradigms.

## 4 Conclusions

In this study, we designed and synthesized iron oxide-based nanochains (FeONCs) for MPI applications. These nanochains showed magnetic susceptibility 4 times higher than conventional SPIONS. Originating from their unique structures, FeONCs showed high sensitivity for MPI and a resolution comparable with that of commercially available MPI tracers. Studies showed that these PEGylated nanochains could target breast cancer via the EPR effect, and RGD-conjugated FeONCs showed augmented tumor accumulation with an enhanced signal intensity, which is 3 times higher than that of the spherical NPs. Future research focused on optimizing these nanoparticles for clinical application will undoubtedly contribute to innovative diagnostic strategies, offering hope for more effective cancer therapies in the years to come. The progress made with FeONCs is not merely a technical achievement; it underscores the evolving landscape of oncology, where science and innovation converge to improve patient outcomes for early detection of breast cancer and hyperthermia-based treatments.

## 5 Experimental Section

### 5.1 Materials

Iron(III) chloride hexahydrate (FeCl_3_·6H_2_O), sodium oleate, oleic acid, 1-octadecene, RITC, 1-ethyl-3-(3-(dimethylamino)propyl)carbodiimide (EDC), ethanol, hexane, chloroform, Dulbecco’s modified Eagle’s medium, Dulbecco’s phosphate-buffered saline, RPMI 1640 medium, penicillin–streptomycin, MTT (3-(4,5-dimethylthiazol-2-yl)-2,5-diphenyltetrazolium bromide), and iron oxide nanoparticles (15 nm) were purchased from Sigma-Aldrich. DSPE-PEG and DPEPEG-NH_2_ were purchased from the NOF American Corporation and Avanti Research, respectively. The Tube-O-DIALYZER mini dialysis system was purchased from Sigma-Aldrich.

### 5.2 Synthesis of Iron Oxide Nanochain

Iron oxide nanochains were prepared as follows by a two-step method.

#### 5.2.1 Synthesis of the Iron–Oleate Complex^47^

For the synthesis of the iron–oleate complex, 10.8 g of iron chloride (FeCl_3_·6H_2_O, 40 mmol) and 36.5 g of sodium oleate (120 mmol) were dissolved in a solvent mixture containing 80 mL of ethanol, 60 mL of distilled water, and 140 mL of hexane. The solution was heated to 70 °C for 4 h. Upon completion of the reaction, the reaction mixture was cooled down to room temperature, transferred into a separatory funnel, and washed with water (3× times), and the hexane layer was collected. Hexane was evaporated, and the product was air-dried to yield an iron–oleate complex as a brown waxy solid.

#### 5.2.2 Synthesis of Iron-Oxide Nanochains (FeONCs)

The as-prepared iron–oleate complex (36 g, 40 mmol) mixed with 5.7 g of oleic acid (20 mmol) was dissolved in 200 g of 1-octadecene at room temperature. The reaction mixture was kept on a heating mantle with a Peltier and was continuously subjected to nitrogen gas to create an inert environment. The reaction mixture was heated slowly using the Peltier with a constant rise of temperature of 10 °C min^–1^ until the desired temperature was reached (300 °C for spherical nanoparticles, 320–330 °C for nanochains, and 340–350 °C for nanocubes). Once the desired temperature is maintained, the reaction is kept at that temperature for 2 h. A vigorous reaction occurs during this process, and the initial transparent solution becomes turbid and brownish for spherical particles, dark coffee color for nanochains, and black for nanocube formation. The resulting solution containing the nanoparticles was then cooled to room temperature, and a desired volume of NPs was washed with a hexane:ethanol (1:3) ratio and separated using a strong magnet. This process repeated until a clear, oil-free particle was obtained.

### 5.3 PEGylation of NPs

The prepared nanoparticles and nanochains were dissolved in chloroform (3 mg/mL). A solution of DSPE-PEG-2000N was prepared in chloroform (30 mg/mL). The two solutions were mixed and sonicated for 1 min. Chloroform was evaporated using reduced pressure and air-dried. Followed by this, distilled water was added to the dried samples to make water-soluble FeONCs and FeONPs. FT-IR and DLS measurements were performed to characterize the nanoparticles.

### 5.4 Preparation of RGD-DSPE-PEG FeONC and FeONP

The prepared DSPE-PEG FeONC/FeONP (5 mg) was mixed with 3 mg of RGD in water, and to this, 10 mg of EDC was added. The reaction mixture was kept at room temperature on a shaker for 12 h. Followed by this, the NPs were dialyzed using a 1000 Da dialysis membrane and were freeze-dried and characterized.

### 5.5 Preparation of RITC-DSPE-PEG- FeONC and FeONP

To prepare RITC-DSPEPEG, RITC (1.5 mg, 0.002 mmol) and DSPEPEG-NH_2_ (7.5 mg, 0.002 mmol) were dissolved in 1 mL of chloroform and stirred overnight. Followed by this, chloroform was removed using reduced pressure and was dialyzed using a 1000 Da dialysis bag, and the product was lyophilized and characterized using FT-IR. For conjugating the RITC-DSPEPEG with nanoparticles, a mass ratio of 1:10 (NPs to RITC-DSPEPEG) was maintained, and the reactants were dissolved in chloroform and sonicated for 1 min. The chloroform was evaporated, and the product was dried, redissolved in water, and characterized using optical absorption spectroscopy and FT-IR studies.

### 5.6 Cell Uptake Experiments

Cell uptake experiments were performed by plating 5 × 10^4^ cells of the desired type (MSC, J774, or 4T1) into each well of a 6-well plate and incubating these cells overnight at 37 °C and 5% CO_2_. The following day, the cells were treated with particles at a concentration of 100 μg of Fe/mL and allowed to incubate overnight, as described above. For the fluorescence-based cellular uptake studies, all the RITC-conjugated NPs/NCs were treated with 4T1 cells at a concentration of 100 μg Fe/mL. After overnight incubation with the particles, cells went through one of two possible detection pathways.

#### 5.6.1 Evaluation of Cell Uptake by Fluorescence

Media was removed from cells by aspiration, and the attached cells were subsequently washed twice in PBS to remove dead cells and any extracellular particles. Then, the cells were stained with DAPI and Calcein-AM for nuclear and membrane identification, respectively. The cells were again washed twice, put in medium without phenol red, and imaged using a Keyence fluorescence microscope.

#### 5.6.2 Evaluation of Uptake by MPI and ICP–OES

Cells had media removed and were washed as described in section a. Following washing, cells were detached by trypsinization and spun down by gentle centrifugation to form a cell pellet. Excess liquid was aspirated off, and the cell pellet was suspended in 1 mL for counting. After counting, 6 × 10^5^, 3 × 10^5^, 1.5 × 10^5^, 6 × 10^4^, 3 × 10^4^, and 1.5 × 10^4^ cells were each placed in separate epi-tubes and were again spun down to remove any remaining liquid. The cell pellets were immediately imaged on the MPI (see below) and then later analyzed by ICP–OES for iron content.

### 5.7 Biocompatibility Study

Cell viability was evaluated using the MTT assay. Briefly, cells are seeded in a 96-well plate and treated with several concentrations of particles, with a no-particle condition serving as a control. The following day, cells are treated with MTT reagent, which is taken up by viable cells and converted to formazan. After 3 h of incubation with the reagent, liquid is removed from the wells, and formazan crystals are dissolved in DMSO. Absorbance is then taken at 570 nm, and all experimental conditions are compared to the untreated control.

### 5.8 MPI and Computed Tomography

MPI (Momentum, Magnetic Insight Inc., CA, USA) was acquired using 2D projection scans or 3D tomographic scans. For 2D imaging, MPI was acquired with default settings (5.7 T/m gradient, 20 mT, 45 kHz excitation) and the following imaging parameters: field of view (FOV) 4 × 6 cm, one average, and an acquisition time of 15 s 3D MPI tomographic scans were performed by using the following parameters: FOV: 12 × 6 × 6 cm, 5.7 T m^–1^ gradient, 16.5 mT (*X*-channel) and 17 mT (*Z*-channel) excitation, 35 projections, and 1 average with an acquisition time of ∼30 min.

Following MPI, CT images were acquired on a Quantum GX micro-CT scanner (PerkinElmer). Whole-body CT images were acquired using 3 × 8 s scans with the following parameters: 90 kV voltage, 88 μA amperage, 72 mm acquisition FOV, and 60 mm reconstruction FOV, resulting in 240 μm voxels.

### 5.9 Image Analysis

2D and 3D MPI data sets were visualized and analyzed utilizing Horos imaging software (Horos is a free and open-source code software program that is distributed free of charge under the LGPL license at https://Horosproject.org and sponsored by Nimble Co LLC d/b/a Purview in Annapolis, MD USA). A calibration graph was prepared for known amounts of Fe content on the *x*-axis and ROI intensities on the *y* axis. From the linear fit of this calibration curve, the amount of Fe was calculated for in vitro and in vivo analysis.

### 5.10 Magnetic Particle Relaxometry

Magnetic particle relaxometry was performed on DSPE-PEG FeONC, RGD FeONC, Vivotrax, Synomag-D50, and Synomag-D70 samples of different Fe masses (5, 10, 25, 50, and 100 μg/mL using 100 μL volume). The signal amplitude (peak signal strength) and spatial resolution (calculated using fwhm) were calculated using the information from the PSF generated using the Origin software.

### 5.11 Animal Models and In Vivo Imaging

All procedures involving animal studies were approved by the Institutional Animal Care and Use Committee (IACUC) of Michigan State University, while animal care and well-being throughout the study were monitored by the Center for Animal Resources (CAR) of Michigan State University. Breast cancer mice were induced by orthotopic injection of 4T1 cells (60,000 cells) into the MFP of 8 week old female NU/J mice (Jackson Laboratory, ME). Cell suspensions were injected into the fourth MFP. Mice were monitored daily for tumor formation by caliper measurement and for body weight for up to 10–12 days. After tumor development, the mice were divided into different groups for the injection of nanoparticles for MPI studies.

### 5.12 Statistical Analysis

Statistical analysis was performed using GraphPad. All data are shown as means ± SDs. Statistical significances were determined using the student’s unpaired *t*-test. *P*-values less than 0.05 were considered statistically significant.

## Supporting Information Available

DLS and zeta potential measurements, FT-IR spectroscopy, cell viability studies, characterization for RITC-based FeONCs, 2D MPI images for the cellular uptake, and ICP–OES measurement for Fe content after cell lysis studies (PDF)
