# Supplementary material for: Development of Iron Oxide Nanochains as a Sensitive Magnetic Particle Imaging Tracer for Cancer Detection
Source: ACS Appl Mater Interfaces. 2025 Mar 26;17(14):20859–71. doi: 10.1021/acsami.5c00332 (PMC11986898; doi:10.1021/acsami.5c00332)
Supplement: Supplementary file 1 — am5c00332_si_001.pdf [file am5c00332_si_001.pdf]

Supporting Information

Development of Iron Oxide Nanochains as a Sensitive Magnetic Particle Imaging Tracer for Cancer Detection

Panangattukara Prabhakaran Praveen Kumar, Md Nafiujjaman, Ashley V. Makela, Kay Hadrick, Meghan L. Hill, Maggie Lee, and Taeho Kim*

Department of Biomedical Engineering, Institute for Quantitative Health Science and Engineering, Michigan State University, East Lansing, MI 48824
*Corresponding Author: kimtae47@msu.edu

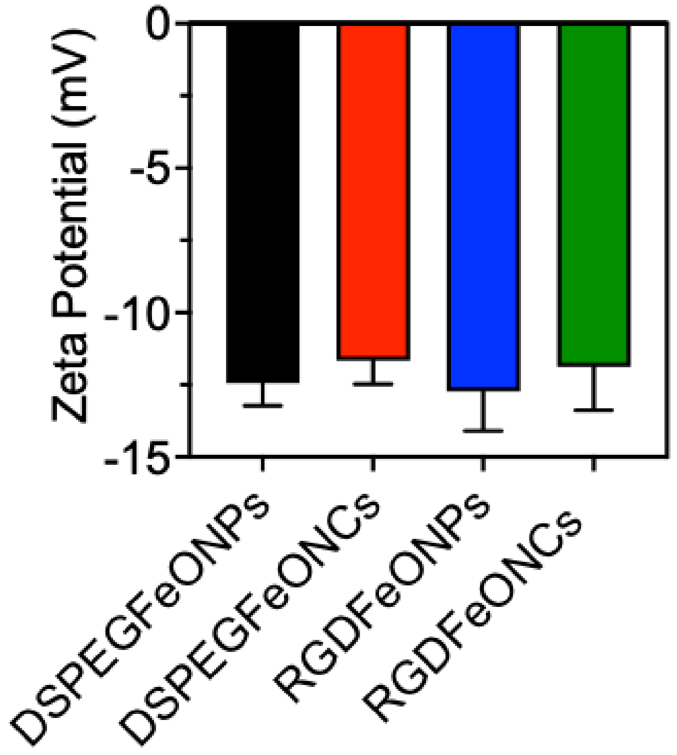

Figure S1. Zeta potential measurements for the FeONCs and FeONPs with various surface modifications (DSPE-PEG and RGD peptides).

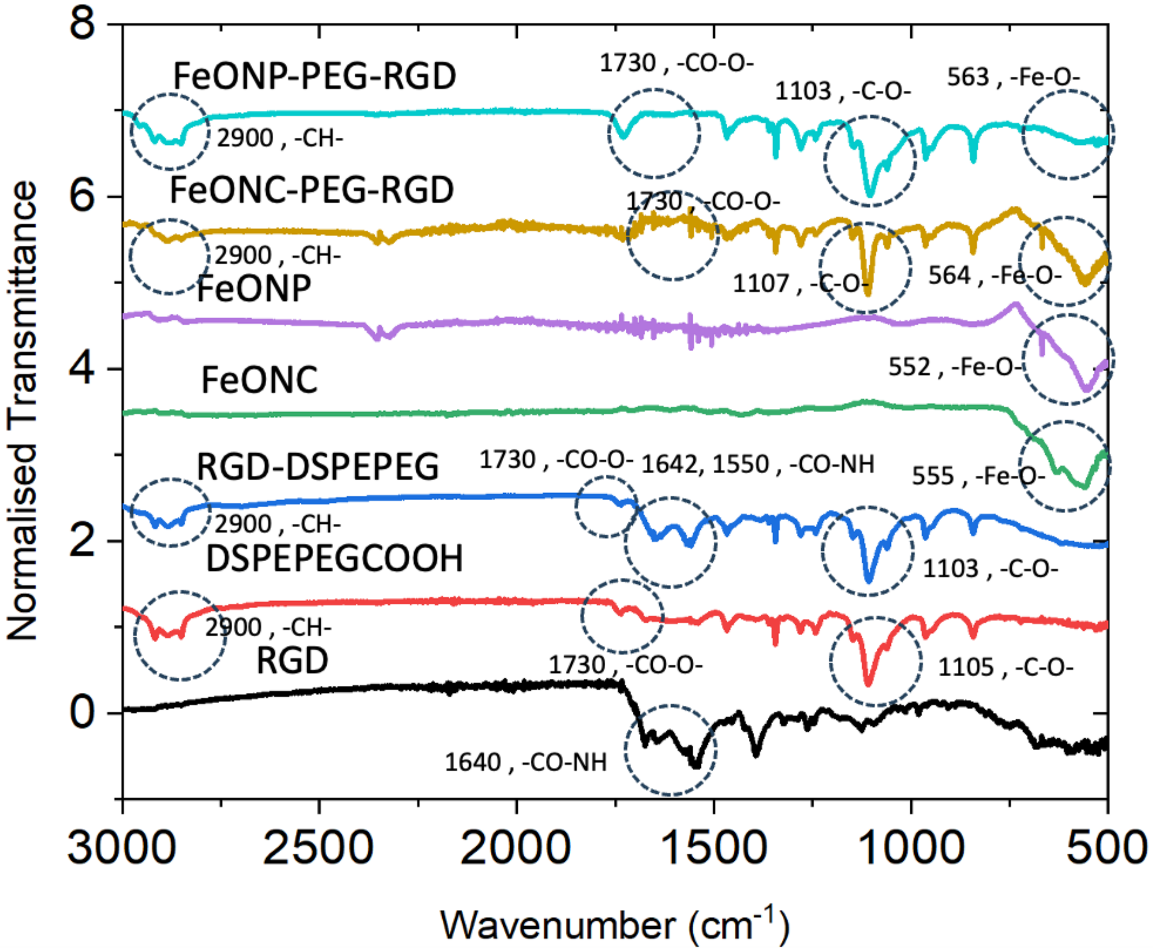

Figure S2. Characterization of prepared iron oxide nanochains (FeONCc) and nanoparticles (FeONPs) with various surface functionalities using FT-IR spectroscopy.

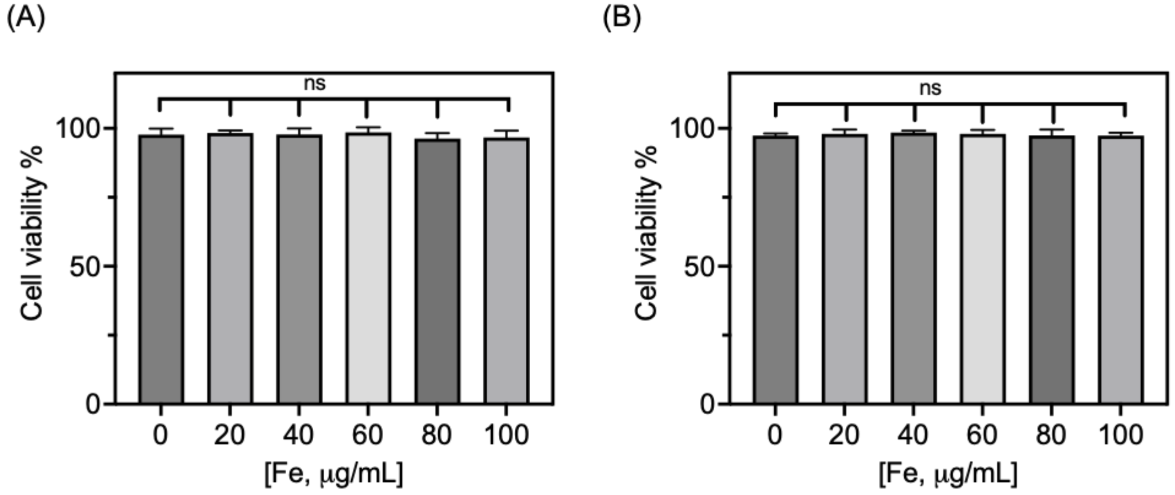

Figure S3. Cell viability by MTT assay for (A) DSPE-PEG FeONCs and (B) RGD FeONCs using mesenchymal cells with varying concentrations of [Fe].

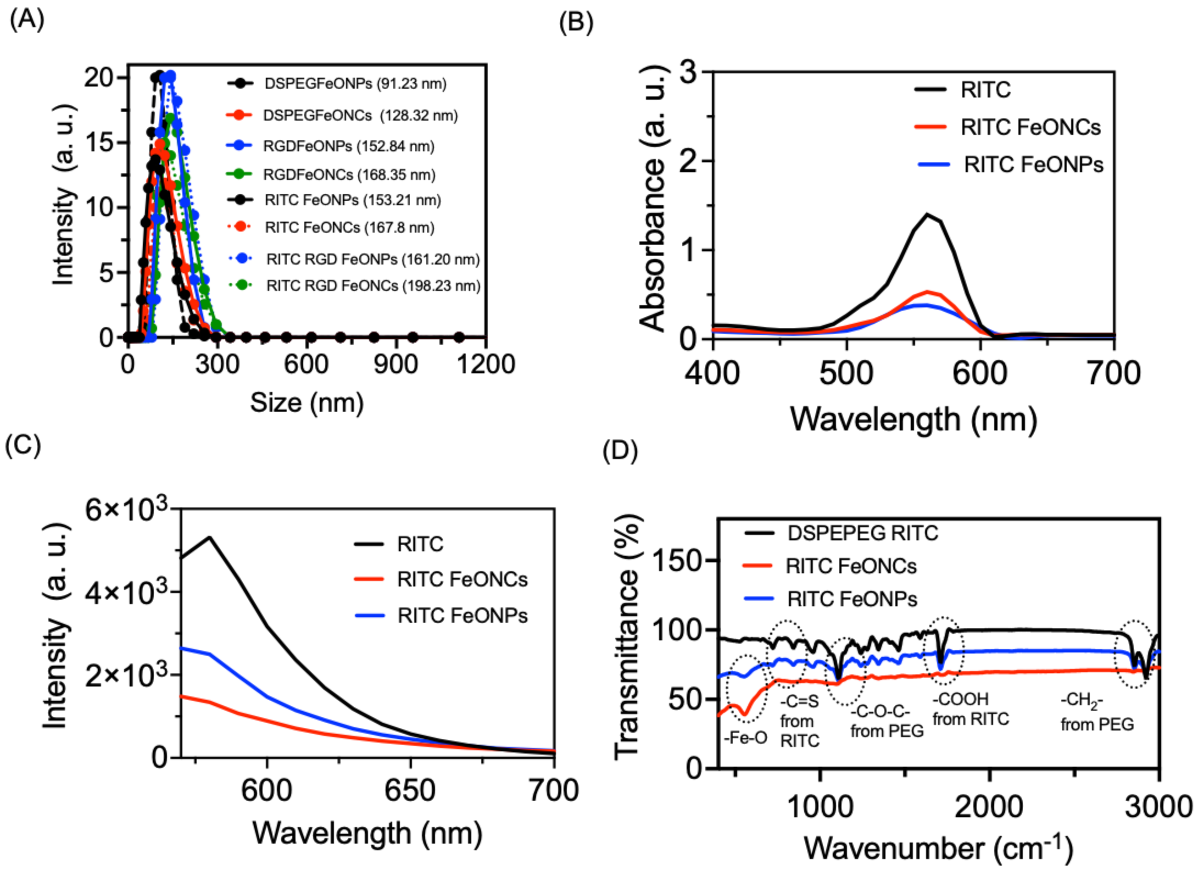

Figure S4. Characterization of RITC FeONC and RITC FeONP. (A) DLS measurement for the NPs. (B) Absorption spectra for RITC (100 µM in DMSO), RITC FeONCs (100 µg/mL in water), and RITC FeONPs (100 µg/mL in water). (C) Emission spectra for RITC (100 µM in DMSO), RITC FeONCs (100 µg/mL in water), and RITC FeONPs (100 µg/mL in water). λexc = 560 nm. (D) FT-IR spectrum for DSPE-PEG RITC, RITC FeONCs and for RITC FeONPs.

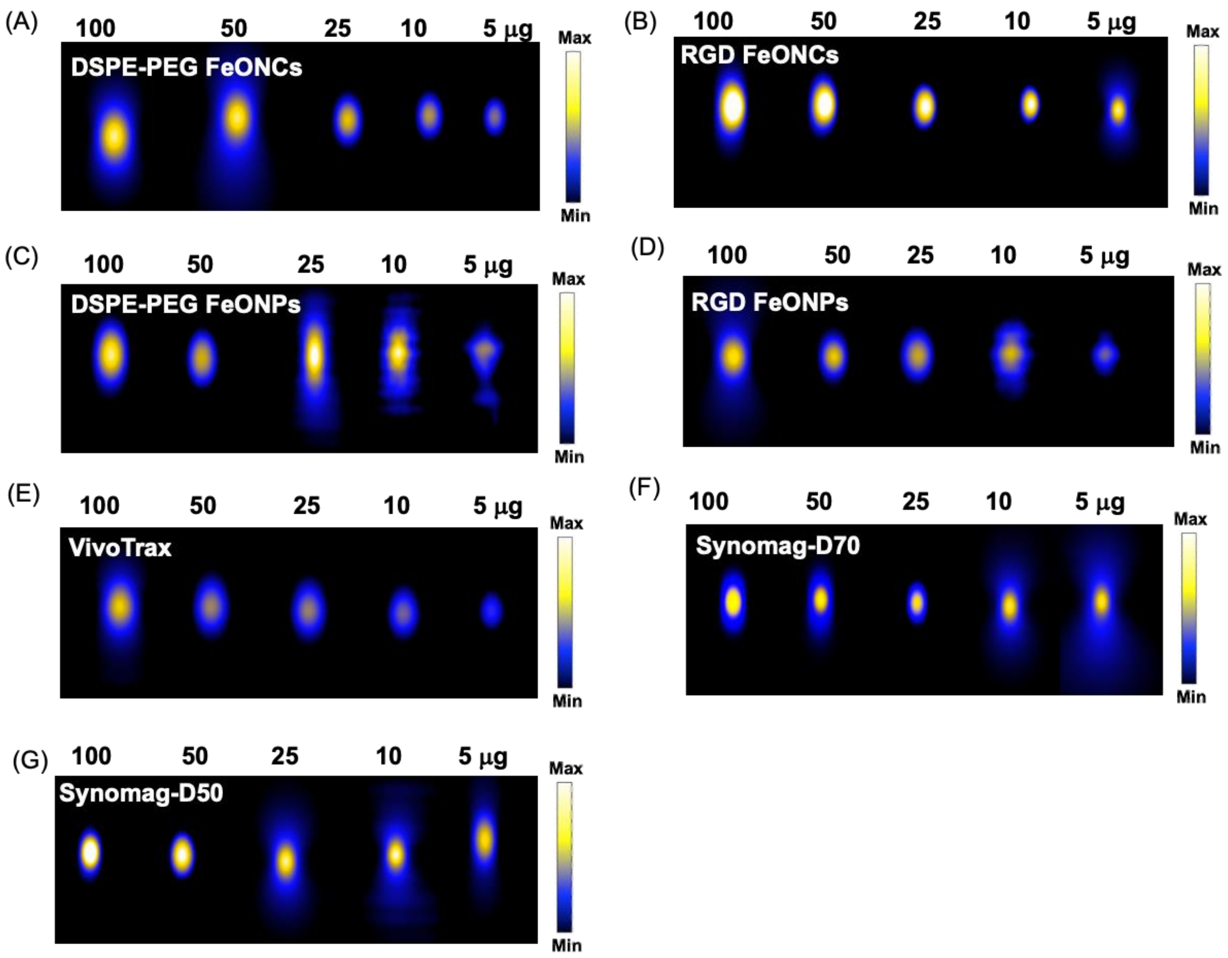

Figure S5. 2D MPI images for various NPs were used for the study. (A-B) represents the 2D MPI images for DSPE-PEG FeONCs and RGD FeONCs, with varying Fe concentrations. (C-D) 2D MPI images for the DSPE-PEG FeONP and RGD FeONP have varying Fe content. (E-G) 2D MPI images for VivoTrax, Synomag-D50 and Synomag-D70 respectively with varying Fe content.

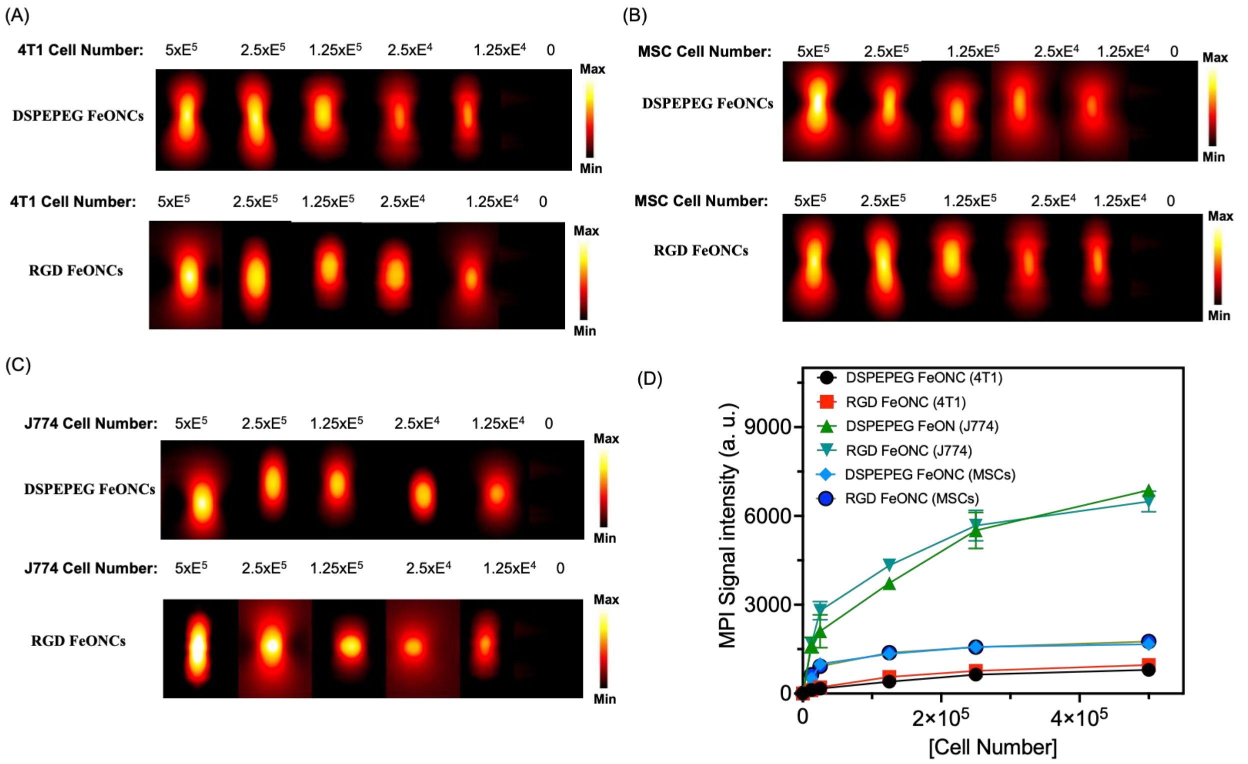

Figure S6. 2D MPI images for the cellular uptake of DSPE-PEG and RGD FeONCs using various cell numbers of (A) 4T1 tumor cells, (B) mesenchymal stem cells (MSC) and (C) for J774 cells, respectively. (D) Quantitative MPI signal intensity from the cell pellets with varying cell numbers.

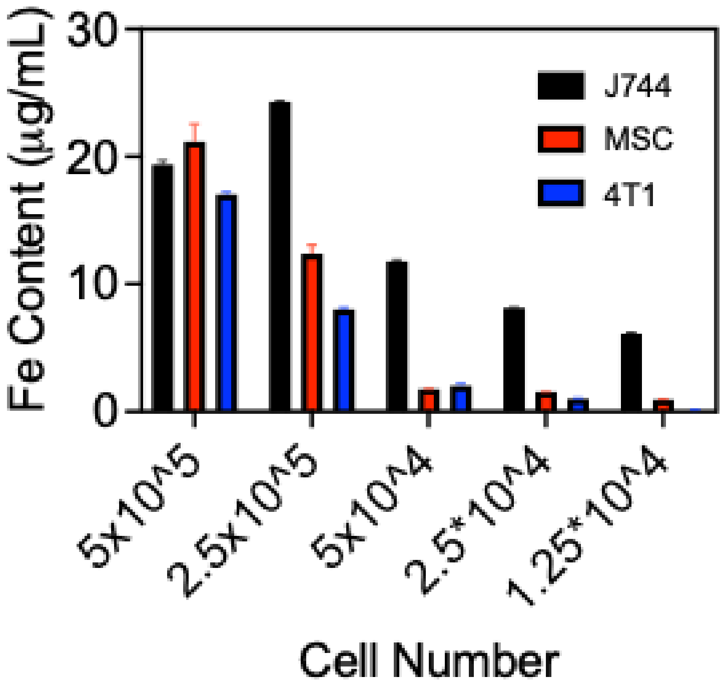

Figure S7. ICP-OES measurements for the Fe content in the cells (J744, MSC, 4T1) after incubating with RGD FeONCs. n = 5.
